# Bilateral brachial synovial cysts in systemic juvenile idiopathic arthritis: Case report and literature review

**DOI:** 10.1111/1756-185X.13618

**Published:** 2019-06-27

**Authors:** Qi Zheng, Meiping Lu

**Affiliations:** ^1^ Department of Rheumatology, Immunology and Allergy Children's Hospital of Zhejiang University School of Medicine Hangzhou China

**Keywords:** brachial synovial cyst, juvenile idiopathic arthritis, subclinical synovitis

## Abstract

**Aim:**

To review the clinical features of brachial synovial cyst.

**Method:**

A case of bilateral brachial synovial cysts is described in a child suffering from systemic juvenile idiopathic arthritis during a relapse. Magnetic resonance imaging and ultrasonography were conducted to further evaluate the nature of the cysts. The case is compared with known cases in a literature review.

**Results:**

Review of the literature showed that brachial synovial cysts occur most commonly in systemic juvenile idiopathic arthritis. It is considered that uncontrolled systemic inflammation and recurrent disease activity might be the cause of synovial cysts.

**Conclusion:**

Brachial synovial cyst is a rare manifestation of juvenile idiopathic arthritis. Uncontrolled systemic inflammation inducing chronic damage to joint structure may be the primary cause of synovial cyst formation.

## 1 INTRODUCTION

Tenosynovitis involving the extensor tendon sheaths on the dorsum of the hand, finger, foot and posterior tibial are frequently seen in children with a polyarticular course of juvenile idiopathic arthritis (JIA). Popliteal synovial cyst is a common occurrence in children with JIA, but cyst formation in other locations, such as brachial synovial cysts are rare.^1^ Brachial synovial cyst is usually unilateral, causing a sudden swelling of the upper arm. Here we describe a child suffering from systemic JIA (sJIA), presenting with bilateral synovial cysts during a relapse of his disease.

## 2 CASE REPORT

A 7‐year‐old boy diagnosed with sJIA 4 years prior to admission, responded to a regimen of prednisone and methotrexate. The dosages of the medications were tapered gradually as symptoms of disease decreased. On follow‐up, he was asymptomatic with normal laboratory tests for 2 years without any therapy.

**Figure 1:**
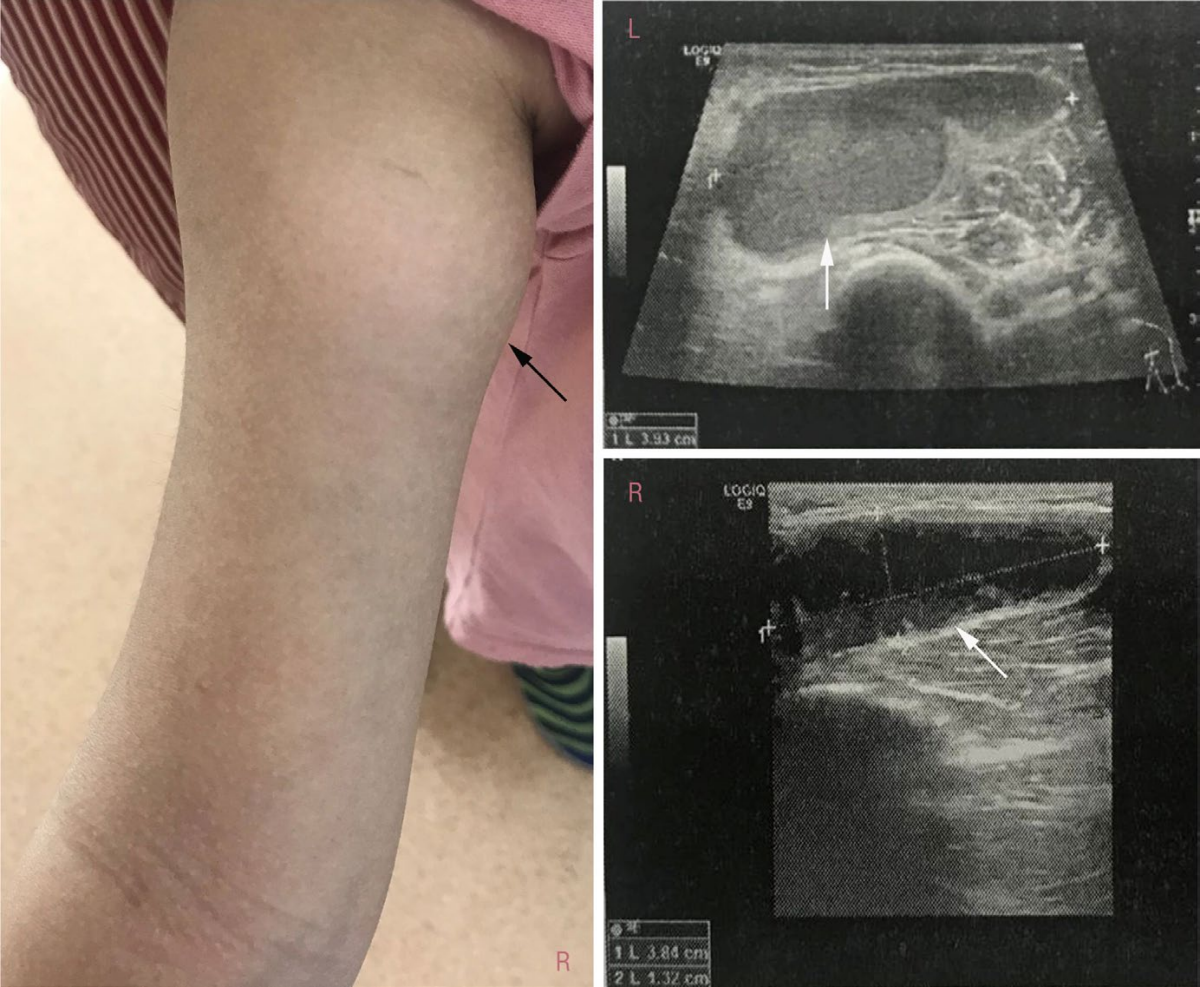
Appearance of bilateral synovial cyst. A, Clinical photograph showing a subcutaneous cyst on right forearm (black arrow). B, Longitudinal view of the cyst with internal septation on left forearm by ultrasonography (white arrow). C, Longitudinal view of the cyst on right forearm by ultrasonography (white arrow)

**Figure 2:**
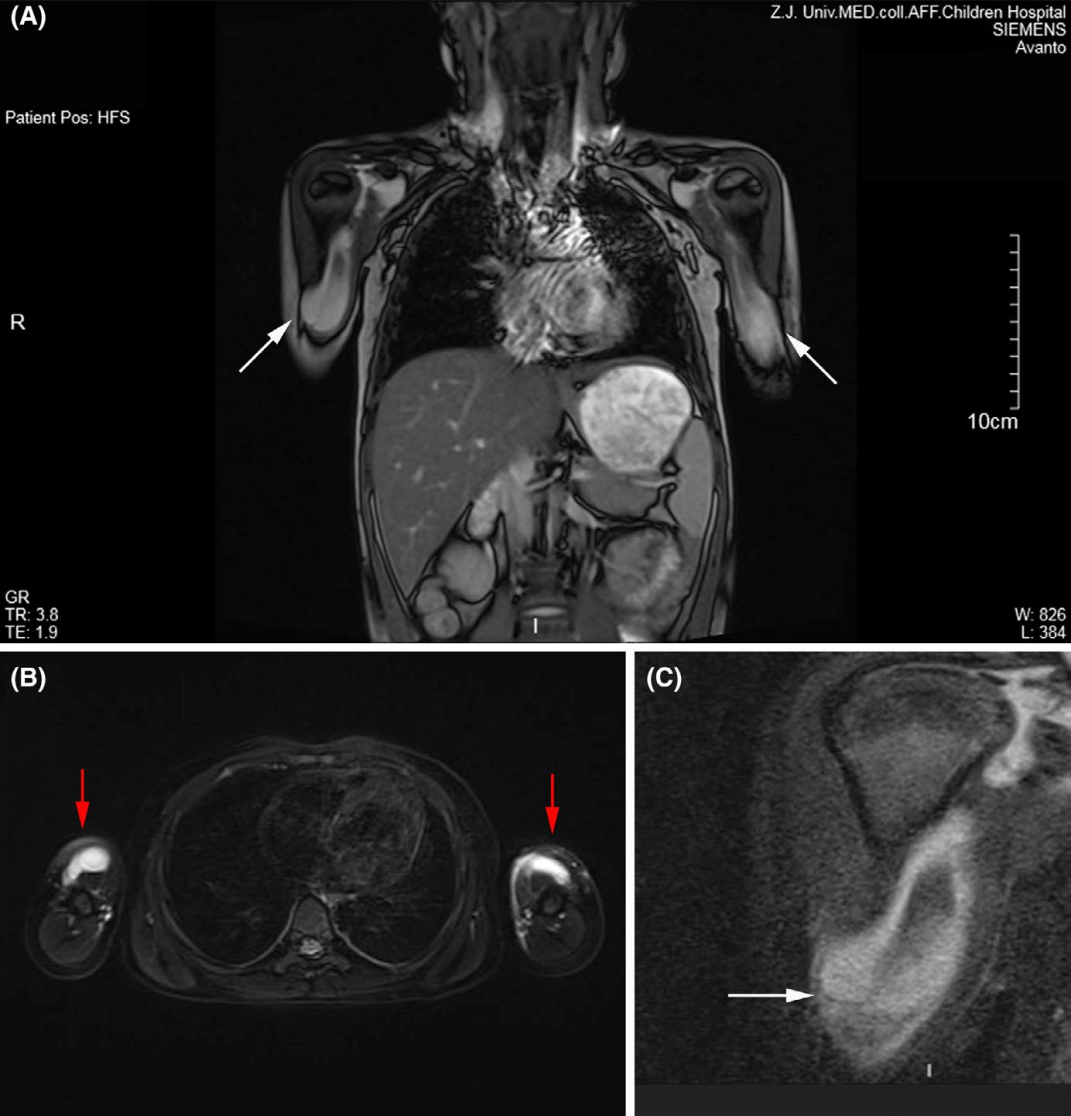
Magnetic resonance imaging (MRI) of bilateral brachial synovial cysts. (A‐C) Longitudinal section of MRI shows irregular cystic fluid signals in bilateral synovial cysts (white arrows). (B) Transverse section of bilateral synovial cysts showing hyperintensity signal on T2‐weighted image (red arrows)

He returned to our hospital on the date of admission with a fever, truncal rash and swollen left ankle. Of note, neither of his shoulder joints had any tenderness or limitation of function at that time. Elevated inflammation markers as well as ferritinemia were found but laboratory tests for infectious pathogens were negative. Other rheumatic diseases were also excluded and therefore it was concluded that this patient had recurrence of his sJIA. He was treated with parenteral methylprednisolone and tocilizumab in accordance with the treatment algorithm for recurrent sJIA. A few weeks later, he developed swelling of his right upper arm, and then the left arm, without redness or pain. At this point, the patient had been treated with three doses of tocilizumab along with prednisone and methotrexate. Ultrasonography (US) displayed cystic mass with internal septation in both arms (Figure 1). When compression was applied with the ultrasound probe, the fluid in the cysts appeared to communicate with the shoulder cavities. In addition, magnetic resonance imaging was performed for further evaluation of the cysts. Irregular cystic fluid signals (hyperintensity on T2‐weighted images) were observed in both shoulder joints, subcutaneous and muscle spaces of the upper arms with internal septation (Figure 2). The cysts were aspirated and cyst fluid from both sides was found to be very viscous with a lot of particulates in it. Bacterial culture was negative. Cell count and other component analyses could not be conducted due to the viscosity of the fluid.

## 3 LITERATURE REVIEW

We then performed a comprehensive review of the literature regarding “brachial synovial cyst” or “bicipital synovial cyst” published in English language journals (Table 1). Literature review in this area demonstrates that nine cases of brachial synovial cyst had been diagnosed since 1980.^2^, ^3^, ^4^, ^5^, ^6^ Of the nine patients, eight were diagnosed as sJIA and one as a sJIA with polyarticular course. Most of these cases were male (7/9). Five in nine patients had recurrent sJIA while the other three patients had disease out of control. All of these patients had unilateral cysts according to the published literature.

**Table 1:** Summary of reported brachial synovial cysts in children

Study First author	No. of cases	Test	Age	Sex	Subtype of JIA	Status
Costello (1980)^6^	1	‐	6	Male	sJIA	‐
Dell'Era (2008)^4^	3	US	6	Female	sJIA	Recurrent
		US	13	Male	pJIA	Recurrent
		US/MRI	15	Male	sJIA	Recurrent
Roth (2006)^5^	2	US/MRI	3	Male	sJIA	Uncontrolled
		US/MRI	5	Male	sJIA	Uncontrolled
Shimizu (2010)^3^	1	US/CT/MRI	4	Female	sJIA	Recurrent
Mizuta (2017)^2^	2	US/MRI	8	Male	sJIA	Recurrent
		US/MRI	12	Male	sJIA	Uncontrolled
Total cases = 9						

Abbreviations: CT, computed tomography; MRI, magnetic resonance imaging; pJIA, poly juvenile idiopathic arthritis; sJIA, systemic juvenile idiopathic arthritis; US, ultrasonography.

## 4 DISCUSSION

The exact etiology of brachial synovial cyst has been a source of controversy. In adults, joint degeneration and complete tear of the rotator cuff with increased fluid production are thought to be the two distinct causes for the formation of synovial cyst.^7^, ^8^ Therefore, repair of the joint capsule through arthroscopy or an open surgical approach is the main therapy for symptomatic patients.

For children, JIA is the only reported association with brachial synovial cyst formation. The reason for the cyst formation is not clear. Pathological findings reveal that the cyst wall is surrounded by granulation and fibrous tissue with abundant inflammatory cell infiltrates, which indicate active inflammation in those patients.^3^ With regard to our patient, we observed joint effusion in the synovial cavities of the shoulders by US, at the time when the disease relapsed but without clinical shoulder joint symptoms. Further, the US examination demonstrated there was a communication between the cysts and synovial cavities of the joints. This suggests that chronic inflammation induced damage to the joint capsule and efflux of the synovial fluid into the bicipital tendon sheath. The cysts were painless without erythema, which indirectly suggested that the formation of brachial synovial cyst was mechanical and non‐inflammatory in nature. Since swelling of the shoulder joint is difficult to ascertain by joint examination, we recommend that major joints be evaluated by US as a part of clinical follow‐up during clinical remission in sJIA patients.

The reason why brachial synovial cyst happens more often in sJIA but not other types of juvenile arthritis is unknown. When treating sJIA, clinicians tend to pay more attention to control of the systemic inflammation than local joint symptoms. Further, clinicians tend to conduct relatively less joint examination than the other types of JIA, such as poly‐JIA or oligo‐JIA during follow‐up. This treatment bias may lead to a false conclusion that local joint symptoms have disappeared when systemic inflammation is under control. For those patient with recurrent sJIA, chronic damage to the joint synovium as well as surrounding structure may be ongoing, even though systemic inflammation is not obvious at that time.^9^, ^10^ This chronic damage could be the reason why brachial synovial cysts are almost always associated with recurrent or uncontrolled sJIA. Joint ultrasonography studies carried out in JIA patients in clinical remission often demonstrate the presence of subclinical synovitis.^11^ Bugni et al^12^ reported that patients with JIA in clinical remission but with positive power Doppler signal on joint ultrasonography, have a higher risk of flare. Therefore, subclinical synovitis should be monitored closely, especially in sJIA patients.

## 5 CONCLUSION

Brachial synovial cyst occurs more often in sJIA than any other type of juvenile arthritis. Uncontrolled systemic inflammation and recurrent disease activity maybe the primary cause of synovial cysts. Subclinical synovitis should be monitored closely by ultrasonography, especially in sJIA patients.

## CONFLICT OF INTERESTS

None.
